# The Resistance to EGFR-TKIs in Non-Small Cell Lung Cancer: From Molecular Mechanisms to Clinical Application of New Therapeutic Strategies

**DOI:** 10.3390/pharmaceutics15061604

**Published:** 2023-05-27

**Authors:** Carmelo Laface, Felicia Maria Maselli, Anna Natalizia Santoro, Maria Laura Iaia, Francesca Ambrogio, Marigia Laterza, Chiara Guarini, Pierluigi De Santis, Martina Perrone, Palma Fedele

**Affiliations:** 1 Medical Oncology, Dario Camberlingo Hospital, 72021 Francavilla Fontana, Italy; feliciamas@gmail.com (F.M.M.); annasantoro86@gmail.com (A.N.S.); iaia.mlaura4@gmail.com (M.L.I.); chiara.guarini@outlook.it (C.G.); pierluigi.desantis1991@gmail.com (P.D.S.); martina.perrone91@hotmail.com (M.P.); 2 Section of Dermatology, Department of Biomedical Science and Human Oncology, University of Bari, 70124 Bari, Italy; dottambrogiofrancesca@gmail.com; 3 Division of Cardiac Surgery, University of Bari, 70124 Bari, Italy; marigialaterza@virgilio.it

**Keywords:** EGFR mutations, non-small cell lung cancer, tyrosine kinase inhibitors, resistance mechanisms

## Abstract

Almost 17% of Western patients affected by non-small cell lung cancer (NSCLC) have an activating epidermal growth factor receptor (EGFR) gene mutation. Del19 and L858R are the most-common ones; they are positive predictive factors for EGFR tyrosine kinase inhibitors (TKIs). Currently, osimertinib, a third-generation TKI, is the standard first-line therapy for advanced NSCLC patients with common EGFR mutations. This drug is also administered as a second-line treatment for those patients with the T790M EGFR mutation and previously treated with first- (erlotinib, gefitinib) or second- (afatinib) generation TKIs. However, despite the high clinical efficacy, the prognosis remains severe due to intrinsic or acquired resistance to EGRF-TKIs. Various mechanisms of resistance have been reported including the activation of other signalling pathways, the development of secondary mutations, the alteration of the downstream pathways, and phenotypic transformation. However, further data are needed to achieve the goal of overcoming resistance to EGFR-TKIs, hence the necessity of discovering novel genetic targets and developing new-generation drugs. This review aimed to deepen the knowledge of intrinsic and acquired molecular mechanisms of resistance to EGFR-TKIs and the development of new therapeutic strategies to overcome TKIs’ resistance.

## 1. Introduction

Non-small cell lung cancer (NSCLC) is the most-frequent cause of cancer-related deaths in the world [1]. Platinum-based chemotherapy was the only therapeutic option for advanced NSCLC patients for many years with a poor prognosis because of a median overall survival (OS) < 12 months [2]. However, the discovery of NSCLC oncogenic drivers led to the development of targeted drugs with an impressive survival benefit for select patients. In particular, the most-important oncogenic drivers are the epidermal growth factor receptor (EGFR) gene mutations [3]. Currently, various EGFR tyrosine kinase inhibitors (EGFR-TKIs) are standard treatment options for patients with activating EGFR gene mutations. 

### 1.1. Epidermal Growth Factor Receptor Pathway in NSCLC 

EGFR (ERBb1/HER1) belongs to the HER (ERBb) family with three other members: HER2 (ErbB2), HER3 (ErbB3), and HER4 (ErbB4) [4]. The binding of specific ligands, such as epidermal growth factor (EGF) and transforming growth factor-α (TGF-α), to the EGFR extracellular domain led to receptor dimerisation with other HER family members [5]. Consequently, the autophosphorylation at the receptor key tyrosine residues takes place. In this way, various downstream signalling pathways are activated including the rat sarcoma (RAS)/rapidly accelerated fibrosarcoma (RAF)/mitogen-activated protein kinase (MAPK) pathway, the phosphatidylinositol-3-kinase (PI3K)/protein kinase B (AKT) pathway, the phospholipase C-protein kinase C (PLC-PKC) pathway, and the janus kinase (JAK)/signal transducer and activator of transcription (STAT) pathway, which regulate cellular proliferation, survival, and apoptosis [6]. EGFR exons 18 to 24 encode the tyrosine kinase domain of the receptor. EGFR-activating gene mutations are located in exons 18, 19, 20, and 21 (the most-common ones in exons 19 and 21) and are responsible for the constitutive EGFR activation, which leads to cell proliferation [7]. These mutations are present in 10–15% of Caucasian NSCLC patients and 30–50% of Asian ones. They regard a more typically adenocarcinoma histotype, women, non-smokers, or Asian NSCLC patients [3].

The EGFR-activating gene mutations in exons 18, 19, 20, and 21 are classically divided into common (exon 19 deletion, exon 21 L858R point mutation), which correspond to 85–90% and generally confer sensitivity to EGFR-TKIs treatment, and uncommon (rare EGFR mutations and complex EGFR mutations), which account for 10–15% and present variable predictive values, from sensitivity to resistance [4,8]. Moreover, it is also possible to find other EGFR alterations consisting of the combination of EGFR mutations with other EGFR mutations or with one or more mutations of other genes (tumour suppressor gene or oncogene). In some cases, only a small percentage of tumour cells has the specific EGFR mutation. The variable sensitivities to EGFR-TKIs could be explained by the variable tertiary structure of the EGFR protein under the influence of the different EGFR mutations [8]. 

Table 1 summarises all categories of EGFR alterations.

#### 1.1.1. Common EGFR mutations

Several studies reported that Del19 (45–50%) appears more frequently than L858R (37–40%) in Asian, as well as in Caucasian populations [4].

The Del19 and L858R mutations lead to elevated receptor dimerisation and activity due to the destabilisation of the inactive conformation of the EGFR receptor [9]. 

While the L858R mutation corresponds to the substitution of leucine to arginine at codon 858 (c.2573T > G, p.L858R), Del19 presents more than 30 variants, and the most-common is delE746_A750 corresponding to 73% of cases, with a deletion of 9 to 24 nucleotides [9]. In 25% of cases, Del19 variants start at position E747. The remaining percentage is known as entitled non-LRE (2%) [5,9].

#### 1.1.2. Uncommon EGFR Mutations

Approximatively 600 EGFR uncommon or rare mutations have been described, accounting for 10–15% of cases, with variable sensitivity to EGFR-TKI treatment [10] and a similar clinical presentation in comparison with common ones. Examples of rare EGFR mutations are some EGFR exon point mutations such as exon 18, G719X; exon 20, S768I; and exon 21, L861Q [4,11].

#### 1.1.3. Other EGFR Alterations

Complex mutations have a prevalence of 5–15% of all EGFR mutations. They are defined by the combination of common and rare EGFR mutations or rare and rare EGFR mutations or common and common ones. The most-frequent EGFR mutations involved in complex mutations are G179X (90%), G709X (75%), and S768I (50%) [12,13,14,15]. The sensitivity to EGFR-TKIs depends on the specific combination of mutations. It is better when one of them is a sensitivity mutation, such as Del19 or L858R, and lower when the combination includes a resistance mutation. For example, E709A + G719C, G787R + L858R, H870R + L858R, and E884K + L858R are sensitive complex mutations, while T790M + L858R is a resistant one [12,13,14,15]. 

Co-mutations correspond to the combination of EGFR mutation(s) with one or more mutations of another gene (tumour suppressor gene or oncogene) [16]. Co-mutations account for similar prevalence across the common EGFR mutations. Their incidence seems to be correlated with prior treatment. These genetic alterations often are found in several genes such as TP53, RB1, CTNNB1 (β-catenin), NKX2-1, or PI3KCA [17,18,19]. Some co-mutations are correlated with a worse prognosis; for example, TP53 mutations, ATM alterations, PTEN-inactivating mutations, KRAS mutations, and IDH1 mutations are associated with lower clinical results following EGFR-TKI treatment [17]. 

Subclonal mutations have a low variant allele frequency (VAF), which may be due to the presence of the specific mutation only in a small percentage of tumour cells [20]. All types of EGFR mutations could be subject to these genetic alterations, in particular the resistant ones. For example, a retrospective analysis of the AURA study, the AURA3 trial, and the study performed by the French Cooperative Thoracic Intergroup showed that the T790 mutation was present only in a small proportion of patients with worse clinical outcomes under third-generation EGFR-TKIs [21,22,23,24].

### 1.2. Clinical Trials

EGFR-TKIs became the standard therapy for advanced EGFR-mutation-positive NSCLC patients after the evaluation of their safety and efficacy in several clinical trials performed in the last decade. Moreover, some clinical studies documented a better prognosis, in terms of progression-free survival (PFS) and OS, for Del19 compared to L858R-mutation NSCLC patients under treatment with EGFR-TKIs [25,26,27]. 

Table 2 summarises all clinical trials evaluating EGFR-TKIs.

#### 1.2.1. First-Generation EGFR-TKIs: Gefitinib, Erlotinib, and Icotinib

The NEJ002 study compared gefitinib versus carboplatin plus paclitaxel as a first-line therapy for advanced NSCLC patients with a common EGFR mutation [28,29].

The IPASS trial investigated gefitinib with carboplatin plus paclitaxel in the same population of the NEJ002 study [30,31].

WJTOG3405 is a phase 3 study in which common-EGFR-mutation NSCLC patients were randomised between gefitinib and cisplatin plus docetaxel [32,33]. 

The OPTIMAL trial evaluated erlotinib with respect to standard chemotherapy as a first-line therapy for common-EGFR-mutation NSCLC patients [34,35].

The ENSURE study analysed erlotinib in comparison with gemcitabine plus cisplatin in Asian patients affected by common-EGFR-mutation NSCLC [36].

In the EURTAC trial, erlotinib was compared to standard chemotherapy as a first-line treatment for European patients affected by common-EGFR-mutation NSCLC [37]. 

The CONVINCE study was designed to evaluate the efficacy and safety of icotinib as a first-line therapy compared to cisplatin/pemetrexed plus pemetrexed maintenance for common-EGFR-mutation NSCLC patients [38].

All these trials reported a significant improvement in terms of PFS, but no statistical difference was seen for OS, maybe because of the high percentage of crossover from standard therapy to the experimental one after disease progression. 

Table 2 summarises all the reported clinical trials.

#### 1.2.2. Second-Generation EGFR-TKIs

The LUX-Lung 3 trial evaluated afatinib versus cisplatin plus gemcitabine or pemetrexed for EGFR-mutation NSCLC patients stratified according to mutation type (exon 19 deletion, L858R, or other) [39].

In the LUX-Lung 6 study, common-EGFR-mutation NSCLC patients were randomised between afatinib versus cisplatin plus gemcitabine or pemetrexed [40].

ARCHER 1050 investigated the safety and efficacy of dacomitinib with respect to gefitinib as a first-line treatment of advanced NCSLC patients with a common EGFR mutation [46].

All these trials reported a significant improvement in terms of PFS, but no statistical difference was seen for OS, maybe because of the high percentage of crossover from standard therapy to the experimental one after disease progression.

#### 1.2.3. Third-Generation EGFR-TKI

AURA3 was designed to evaluate the safety and efficacy of osimertinib in comparison with cis/carboplatin plus pemetrexed for advanced NSCLC patients who experienced disease progression after first-line EGFR-TKI therapy and developed the EGFR T790 mutation. Indeed, osimertinib is the third-generation TKI selective for T790M resistance mutations. The authors reported a longer PFS (10.1 versus 4.4 months; HR 0.30; *p* < 0.001) and OS (26.8 versus 22.5 months; HR 0.87, *p* = 0.277) for the osimertinib group, although the latter had no significant difference. This is probably due to the high crossover rate from chemotherapy to osimertinib of patients with progressive disease. After crossover adjustment, there was an HR of 0.54 for OS. The ORR was significantly better with osimertinib (71%) than the control group (31%) (odds ratio for OR: 5.39; *p* < 0.001). Encephalic PFS was also significantly longer for patients treated with osimertinib (8.5 months vs. 4.2 months; HR 0.32) [42]. 

The FLAURA trial tested osimertinib versus standard EGFR-TKIs (gefitinib or erlotinib) in previously untreated patients with common-EGFR-mutation NSCLC. The results reported PFS and OS significantly longer for the osimertinib group (PFS: 18.9 months vs. 10.2 months; HR 0.46; *p* < 0.001. OS: 38.6 months vs. 31.8 months; HR 0.80 *p* = 0.046). The ORR was similar (80% with osimertinib and 76% with standard EGFR-TKIs; odds ratio: 1.27; *p* = 0.24) [26]. As a consequence of the good results reported in this trial, osimertinib has become the first-line treatment for advanced or metastatic EGFR-mutant-positive NSCLC, regardless of T790M status.

The ADAURA study analysed osimertinib for 3 years as an adjuvant therapy for NSCLC patients with stage IB-IIIA and common EGFR mutations who have or have not previously received adjuvant chemotherapy. The DFS rate was 73% and 38% at 4 years (HR 0.27; *p* < 0.001) for the overall population in the osimertinib and control group, respectively. As regards CNS disease, at 24 months, 98% and 85% of patients in the experimental and placebo group were alive and did not have central nervous system disease (HR 0.18). The OS results are still immature [43].

#### 1.2.4. EGFR-TKIs Specific for Ins20 

ZENITH20-2 is a multicentre, multicohort, open-label phase 2 trial that investigated poziotinib for previously treated advanced NSCLC patients with EGFR exon 20 insertions that demonstrated resistance to approved TKIs. This type of mutation is an oncogenic driver and accounts for 2–5% of NSCLCs. The ORR was 27.8%. The disease control rate (DCR) was 70.0%, and PFS was 5.5 months [44].

Mobocertinib was tested in a phase 1/2, dose-escalation and dose-expansion trial that enrolled pretreated patients with advanced NSCLC and EGFR exon 20 insertions. This drug is a TKI targeting EGFR exon 20 insertions in NSCLC. The ORR was 43%, and PFS was 7.3 months [45].

#### 1.2.5. EGFR-TKI Treatment Combinations as First-Line Therapy

Therapeutic approaches based on treatment combination have been designed with the aim to delay cancer progression by limiting the heterogeneity of resistance mechanisms. In this regard, the combination of an EGFR-TKI (afatinib) with an anti-EGFR antibody (cetuximab) has been evaluated in both preclinical and clinical data with interesting results. In detail, this approach demonstrated overcoming the resistance correlated with the T790M mutation by inducing a degradation of EGFR. On these bases, IFCT-1503 ACE-Lung, a randomised and open-label phase 2 study, has been designed to compare afatinib plus cetuximab versus the afatinib single agent as a first-line therapy of advanced EGFR-mutant NSCLC. However, the study ended early after a futility analysis documented the comparable results between the two groups [47].

There is strong biologic rationale for therapeutic approaches targeting both the vascular endothelial growth factor (VEGF) and EGFR pathways in NSCLC because they are closely related to each other. In detail, VEGF is known to be downregulated by EGFR inhibition through hypoxia-inducible factor-1alpha-dependent and -independent mechanisms. In addition, acquired resistance to EGFR-TKIs is correlated with increased levels of VEGF, and dual-VEGF/EGFR inhibition has demonstrated activity in the case of EGFR-TKI-resistant disease [48,49,50,51]. To this end, some clinical studies evaluated this type of combination. BELIEF is a multicentre, single-arm, phase 2 trial that investigated the safety and efficacy of erlotinib plus bevacizumab for advanced NSCLC patients with common EGFR mutations. Moreover, the enrolled patients were stratified according to the pretreatment presence of the T790M mutation to test the hypothesis that the coexistence of the T790M resistance mutation with another EGFR mutation correlates with a shorter PFS. The overall PFS was 13.2 months; however, the primary endpoint was met only for T790M-positive patients (PFS of 16 months), unlike the T790M-negative group (PFS of 10.5 months) [52]. 

JO25567 is a multicentre, open-label, randomised, phase 2 study that evaluated the erlotinib single agent or with bevacizumab as a first-line treatment in patients with advanced NSCLC and common EGFR mutations. PFS was 16 months in the experimental group and 9.7 months in the control one (HR 0.54, *p* = 0.0015). No significant benefit in OS was observed [53].

RELAY is a randomised, double-blind, placebo-controlled, phase 3 trial that compared erlotinib plus ramucirumab with erlotinib monotherapy in patients suffering from untreated common-EGFR-mutated advanced NSCLC and no CNS metastases. PFS was significantly longer in the experimental group (19.4 months versus 12.4 months; HR 0.59, *p* < 0.0001). The OS data are still immature [54]. Preclinical data about osimertinib in association with chemotherapy (pemetrexed or cisplatin) reported a delay in the occurrence of acquired resistance and long-lasting effects, even after treatment discontinuation. However, efficacy was lower when osimertinib was administered before chemotherapy; a possible explanation could be that the EGFR-TKI-induced G1-phase blockade protects cells from chemotherapy toxicity. Wu et al., in a meta-analysis, investigated the efficacy and safety of EGFR-TKI plus chemotherapy compared to EGFR-TKI alone as a first-line treatment in advanced NSCLC patients with the common EGFR mutation. The combination and concurrent therapy groups experienced a significantly higher OS (*p* = 0.02 and *p* = 0.002, respectively) [55]. 

FLAURA2 is a phase 3 trial that is currently testing the combination of chemotherapy with osimertinib [56].

Table 3 summarises all clinical trials evaluating EGFR-TKI treatment combinations as first-line therapy.

## 2. Mechanisms of Resistance

**Figure 1:**
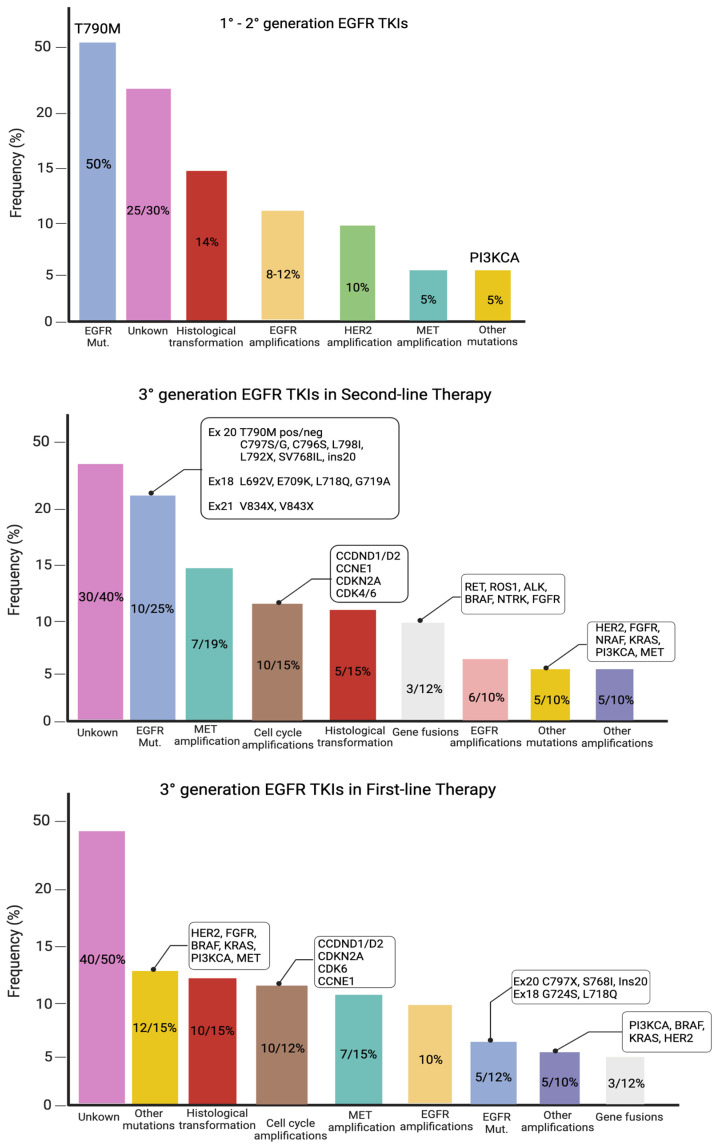
Mutations of resistance to EGFR-TKIs, according to the generation of TKIs and the line of therapy.

**Figure 2:**
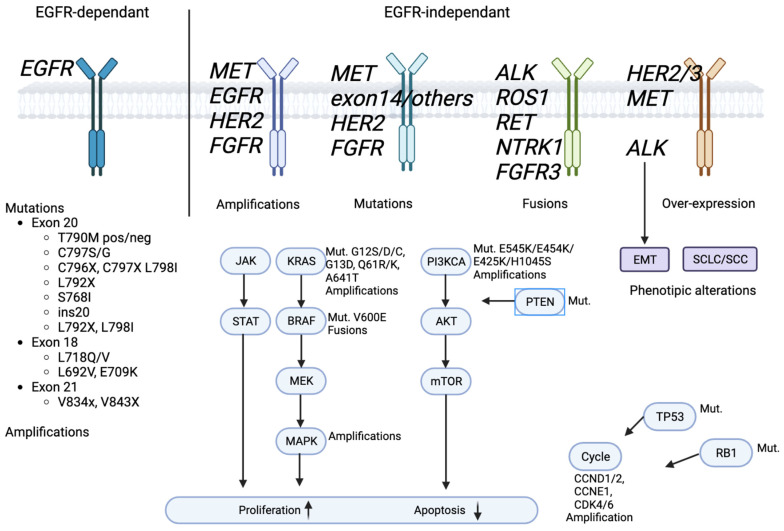
EGFR signaling pathway and EGFR-TKIs’ resistance mechanism.

Although all generations of EGFR-TKIs have been proven to be very effective for NSCLC with common EGFR mutations, almost 5–25% of these patients do not experience a clinical benefit with these drugs due to intrinsic resistance [57,58]. On the other hand, the major part of patients treated with EGFR-TKIs became resistant to these therapies despite an initial response or stable disease. The various mechanisms of resistance to EGFR-TKIs could be explained by the high molecular heterogeneity of NSCLCs (Figure 1 and Figure 2) [59]. Therefore, deepening the knowledge about the EGFR-TKI resistance mechanisms is one of the most-important aims in order to improve the treatment strategy of these patients. 

### 2.1. Intrinsic Resistance

Patients with intrinsic resistance report an early tumour progression without prior tumour response; some of them respond for a very short period (<3 months) [60].

A possible cause regards the pharmacokinetics. In detail, treatment can fail due to the ineffective drug dose in the target area. This event can occur because of drug competition or the difficulty of first-/second-generation EGFR-TKIs to reach sanctuary localisations, such as the brain [61]. However, intrinsic resistance is often due to the lack of a target dependency (i.e., EGFR exon 20 mutations) or the genes alterations from other pathways (downstream or parallel pathways) [62,63]. Mechanisms of intrinsic resistance have been reported above all in patients with uncommon mutations and, more rarely, with common ones [62,63]. This type of resistance often depends on the presence of a drug-resistant EGFR mutation; the most-important ones are the exon 20 insertions (1–10% of all EGFR mutations) and the T790M EGFR mutation (approximately <1–65% of cases, based on the detection method employed) [64,65,66,67]. The lack of first-/second-generation EGFR-TKIs’ efficacy for the T790M mutation and exon 20 insertions is the reason for the development of third-generation EGFR-TKIs and EGFR-Ins20-specific inhibitors, respectively.

#### 2.1.1. Intrinsic Resistance to First-/Second-Generation EGFR-TKIs

Exon 20 insertions correspond to the addition of residues at the N-lobe of EGFR (M766 to C775), while the C-helix (A767 to C775) is their preferential location [68]. This area is fundamental to regulate ATP and EGFR-TKI binding with the consequent activation of the kinase domain through a conformation change [68]. Commonly, exon 20 insertion mutations led to a reduced sensitivity to EGFR-TKIs; however, in vitro studies described that some insertion mutations, such as the insertion EGFR-A763_Y764insFQEA, confer high sensitivity to EGFR-TKIs [69,70]. These data have been confirmed by clinical trials in which NSCLC patients with some types of insertion mutations experienced prolonged periods of disease control under EGFR-TKI treatment [69]. 

The presence of the EGFR T790M mutation at diagnosis is a rare event, which suggests, in some cases, a germinal EGFR mutation [66,67,71]. It is associated with the worst response to first- or second-generation EGFR-TKIs and poor clinical outcomes [66,67,72]. The EGFR T790M mutation regards exon 20 and consists of a substitution of the threonine at position 790 with a methionine in the ATP-binding pocket of the kinase domain. This change prevents the binding of EGFR-TKIs to the receptor due to a steric hindrance; on the other hand, it leads to an in increased affinity between ATP and EGFR. Therefore, the receptor affinity for ATP becomes greater than that for the drug with a severe reduction in EGFR-TKI activity [73].

Another EGFR mutation that is responsible for intrinsic resistance to EGFR-TKIs in vitro is the variant III (vIII) in-frame deletion of exons 2–7 in the extracellular domain [74]. This mutation is present in almost 5% of human lung squamous cell carcinoma and determines the unsuccessful binding of EGF and other growth factors to EGFRvIII [75,76]. The reason behind the constitutive activation of EGFRvIII and the EGFR-TKI resistance is probably the structural changes of the EGFR protein affecting the ATP pocket and the intracellular domain conformation [74].

Some genetic alterations could occur in NSCLC patients with common EGFR mutations with the consequent reduction of sensitivity to EGFR-TKI therapy. In this regard, BIM is a proapoptotic member of the Bcl-2 family that plays a critical role in apoptosis mediated by EGFR-TKIs [77]. NSCLC patients with deletion polymorphisms or low-to-intermediate levels of BIM mRNA have poor clinical efficacy under EGFR-TKIs [77,78]. Furthermore, low levels of NF1 and the overexpression of RhoB are correlated with poor clinical efficacy [79,80]. Moreover, the plasma detection of TP53 gene co-mutations within two months of EGFR-TKI treatments is related to the worst PFS and OS [81]. CRIPTO1 is a member of the EGF-CFC family; it is a cell membrane protein linked to glycosylphosphatidylinositol. High basal levels of CRIPTO1 lead to a reduced EGFR-TKI sensitivity through the activation of ZEB1 and SRC. In this way, ZEB1 promotes epithelial-to-mesenchymal transition (EMT), while SCR stimulates AKT and MEK signalling [82]. 

The major part of oncogenic driver mutations in NSCLC is mutually exclusive, although some of them are present simultaneously, such as PI3KCA or TP53 mutations, with some other oncogenic driver mutations [83,84]. If, on the one hand, co-mutations of the PI3KCA and EGFR genes have no clinical impact [83], on the other hand, the co-mutation of EGFR Del19 and non-disruptive TP53 exon 8 is correlated with intrinsic resistance to first-generation EGFR-TKIs [84].

In some cases, pretreatment AXL and CDCP1 RNA overexpression coexist with EGFR mutations and correlate with poor response to first-generation EGFR-TKIs [85] and, likewise, co-alterations in some cell cycle genes or genes of the PI3K, MAPK, and Wnt/β-catenin pathways [16,60]. 

#### 2.1.2. Intrinsic Resistance to Third-Generation EGFR-TKIs

Although most studies regard resistance to osimertinib during second-line therapy, some literature data report intrinsic resistance when it is administered as a first-line treatment [86]. In this regard, the transformation of NSCLC to SCLC has been considered a possible mechanism of intrinsic resistance [87]. 

The HER2 and MET genes’ amplification was associated in in vitro studies with reduced sensitivity to third-generation EGFR-TKIs such as osimertinib and rolecitinib [88,89]. The combination of the KRAS G12D mutation and PTEN loss was also detected in NSCLC patients with intrinsic resistance to osimertinib [60]. The worst response to third-generation EGFR-TKIs was reported also in NSCLC patients with the EGFR mutation and CDCP1 or AXL RNA overexpression at baseline [85].

### 2.2. Acquired Resistance

All the patients treated with EGFR-TKIs experience a progression of disease (PD) after a variable period of treatment. Patients usually develop acquired resistance after 9–12 months of treatment with first-/second-generation EGFR-TKIs, almost 10 months with second-line third-generation EGFR-TKIs [42], and about 19 months with first-line third-generation EGFR-TKIs [27].

Clinical criteria of acquired resistance to EGFR-TKIs in NSCLC patients have been proposed by Jackman et al., although further clinical validation is needed. The criteria by Gandara et al. are based on the type of PD: central nervous system (CNS), systemic, and oligo-progression. In the consideration of the undefined management of NSCLC patients who progressed to EGFR-TKIs because of acquired resistance, this classification could help clinicians establish the best treatment strategy based on the PD patterns [88]. For example, the same treatment with EGFR-TKI could be continued in patients with slow-PD and without clinical deterioration. A similar strategy could be applied for those patients with CNS PD or oligo-PD in association with local treatment to the site of progression (e.g., radiotherapy or surgery) [90].

The comprehension of the mechanisms leading to the acquired resistance is complex due to different aspects such as: (1) the type of EGFR-TKI; (2) the line of treatment with a specific EGFR-TKI; (3) the tumour biology, in particular histology, intrinsic mutability, microenvironment, and the type of initial EGFR mutation. 

In the literature, there are several studies regarding the development of acquired resistance to the first-line treatment with first- or second-generation EGFR-TKIs or second-line treatment with third-generation EGFR-TKIs, usually due to the occurrence of the T790M mutation. In contrast, few data have been published about the acquired resistance to first-line osimertinib treatment. However, given the increasingly larger number of patients who will be treated with this drug, it is crucial to deepen the knowledge about the biological mechanisms of EGFR-TKI resistance.

Below, we describe the acquired resistance mechanisms to EGFR-TKIs known today. In detail, they can be classified into EGFR-dependent due to the insurgence of new EGFR mutations and EGFR-independent mechanisms due to the activation of alternative pathways.

#### 2.2.1. EGFR-Dependent Mechanisms: Secondary EGFR Mutations 

Acquired resistance based on EGFR-dependent mechanisms is due to the insurgence of secondary and tertiary mutations and/or amplifications of the EGFR gene with the consequent alteration of the receptor aminoacidic structure. Therefore, this leads to a conformational change that can regard the kinase or the ATP-binding pocket of the mutant EGFR, limiting drug accessibility or increasing the ATP affinity.

The incidence of EGFR-dependent acquired resistance is variable based on the type of EGFR-TKI administered and the line of treatment. To be specific, approximately 50% of patients develop this type of resistance under first-/second-generation EGFR-TKIs, 20% of them if they are treated with third-generation EGFR-TKI as a second-line therapy, and 10–15% with first-line third-generation EGFR-TKIs [91]. 

##### EGFR T790 Mutation

This is the most-frequent (49–63%) secondary mutation resulting in the insurgence of acquired resistance under treatment with first-/second-generation EGFR-TKIs [92]. Therefore, third-generation inhibitors were specifically designed to target the EGFR T790 mutation.

The EGFR T790M mutation regards exon 20 and consists of a substitution of the threonine at position 790 with a methionine in the ATP-binding pocket of the kinase domain. This change prevents the binding of EGFR-TKIs to the receptor due to a steric hindrance; on the other hand, it leads to an increased affinity between ATP and EGFR. Therefore, the receptor affinity for ATP becomes greater than that for the drug with a severe reduction in EGFR-TKI activity [93].

Some studies have hypothesised that this type of resistance might depend on the selection of pre-existing drug-resistant EGFR-T790M-positive clones during treatment with first-/second-generation EGFR-TKIs or on de novo acquisition of the EGFR-T790M mutation by initially drug-tolerant cells, negative for the EGFR T790M mutation [94]. Experimental data on gefitinib-resistant PC9 cells showed that the early EGFR T790M mutant clones derived from pre-existing EGFR T790M mutated cells were selected for gefitinib treatment. The other theory includes the late de novo occurrence of this type of mutation in drug-tolerant cells due to the prolonged exposure to a first-/second-generation EGFR inhibitor [64]. In an in vitro study, Hata et al. documented the restorations of late-emerging T790M cells’ sensitivity to third-generation EGFR-TKIs thanks to the treatment of tumour cells with navitoclax, an inhibitor of the antiapoptotic factors BCL-2 and BCL-xL [95]. 

Approximately 43% of NSCLC patients lose the EGFR T790M mutation with the PD [30,64,68]. This event suggests the existence of subclones with the EGFR T790M mutation [88]. Usually, the loss of T790M is associated with the presence of exon 19 deletion (83%) and, only rarely, with the L858R mutation (14%) [68]. Moreover, from a clinical point of view, this event at the time of progression is correlated with the worst clinical outcomes [23,96,97,98]. From a molecular point of view, it is associated with the loss of EGFR dependence and dependence on non-EGFR mechanisms [96]. 

##### Tertiary EGFR Mutations: Resistance to Second-Line Third-Generation EGFR-TKI

The AURA3 trial was the first study that showed the insurgence of acquired resistance to second-line osimertinib by means of the employment of cell-free DNA (cfDNA) genomic profiles [96,99]. To be specific, the results demonstrated that about 50% of the NSCLC patients maintained the EGFR T790M mutation, including those that experienced the insurgence of the tertiary EGFR mutation. The authors reported that acquired tertiary EGFR mutations occurred in 21% of cases, and the most-common one (15%) was the EGFR exon 20 C797S mutation [42,100,101]. In contrast, the FLAURA study, in which osimertinib was administered as a first-line therapy, reported a C797S mutation frequency of 7%.

The C797S mutation corresponds to a substitution in the ATP-binding site of a cysteine with a serine at codon 797, resulting in the inability of osimertinib to covalently bond with the mutant EGFR [102]. Moreover, some studies showed that this mutation also prevents the binding of other irreversible third-generation EGFR-TKIs such as olmutinib, rociletinib, and narzatinib to the EGFR active site [103,104]. Interestingly, the allelic context of the C797S mutation can predict the response to subsequent EGFR-TKI therapies. In detail, when NSCLC patients have the T790M and C797S mutations on the same allele (*cis*-mutations), they experience resistance to all available generations of EGFR-TKIs as a single agent or combined with other drugs [104,105]. On the other hand, when patients have these mutations on different alleles (*trans*-mutations), they experience sensitivity to first- and second-generation EGFR-TKIs. However, mutations in trans are rare, regarding less than 30% of cases [105,106]. 

Rare point EGFR mutations in exon 20 have been also identified in the C796 residue such as the G796R (0.56% of patients with lung adenocarcinoma treated with osimertinib), G796S, and G796D mutations, which are adjacent to C797 in exon 20 and can sterically impair the binding of osimertinib to EGFR [103,107,108,109]. 

L792 exon 20 mutations, including L792H, L792Y, and L792F, consist of the addition of a benzene or imidazole ring to the side chain of L792, resulting in the binding disruption of osimertinib to the EGFR kinase domain [108,110]. This mutation usually is located in cis with T790M, but less frequently, it can also occur in trans with EGFR C796/C797X mutations [105].

The L718Q, L718V, and L798I mutations in exon 18 affect the ATP-binding site of the EGFR kinase domain. Therefore, they determine steric restriction, preventing the binding of osimertinib [97,105,110]. These mutations are responsible for osimertinib resistance independent of the C797 mutation; in fact, they are not co-existent. L718Q/V are associated with sensitivity to first- and second-generation EGFR-TKIs, above all when T790M has been lost [111]. Osimertinib resistance is also caused by the G719A mutation located close to the L718 residue [105].

G724S is a very rare EGFR mutation located in exon 20, usually associated with EGFR exon 19 deletion. This mutation regards the P-loop of the kinase domain interfering with the binding of osimertinib [112,113,114]. However, this altered structure does not confer resistance to second-generation EGFR inhibitors [62]. 

SV768IL (S768I + V769L) is another rare (3%) mutation of EGFR exon 20 that has been identified in second-line therapy with osimertinib [101]. 

Rarely, tertiary EGFR mutations such as G724S, L718Q, V834L, and L718V can occur in patients that lost the T790M mutation [97]. 

##### Secondary EGFR Mutations: Resistance to First-Line Third-Generation EGFR-TKI

FLAURA was the first study that evaluated resistance to osimertinib as a first-line treatment [27,115]. Other literature data derive from some case reports or small case series [96,116]. 

This phase 3 trial analysed cfDNA samples through NGS, but no emergent T790M mutation has been detected. This discovery is in line with the well-known activity of osimertinib towards EGFR-sensitising and T790M mutations [115]. In this study, EGFR mutation/amplification was rare (9%), as well as C797S mutation frequency (7%), although it is the most-common mechanism after MET amplification (15%) [101]. S768I or combined EGFR mutation, such as Del19 + G724S (exon18), L718Q + EGFR ex20ins (exon 18 + 20), C797X, or S768I (exon 20), or L718Q + C797S, L718Q + L797S (exon 18 + 20) are very rare secondary mutations, each corresponding to about 1% of cases [11,12,115,117].

Interestingly, this study gave evidence that the mechanisms of resistance to first-line osimertinib depend on EGFR only for a small proportion of cases, and no EGFR T790M mutation was observed. Alternatively, EGFR-dependent mechanisms of acquired resistance are typical for those patients who receive osimertinib as a second-line therapy. Therefore, the T790M mutation will be less common due to the more-frequent use of osimertinib as the first-line therapy.

##### Rare EGFR Mutations 

Although the underlying mechanisms are not well-defined yet, literature data described other rare EGFR point mutations that are responsible for acquired resistance to first-/second-generation EGFR-TKIs and regard less than 10% of NSCLC patients. They include D761Y and L747S (exon 19) or T854A (exon 21), Asp761Tyr, 39 Thr854Ala, and 40 Leu747Ser [71,110,118]. 

Other rare molecular alterations, such as the β-catenin mutation, have been detected in association with the EGFR T790M mutation [92].

#### 2.2.2. EGFR-Independent Mechanisms: Alternative Pathways

##### Oncogene Amplification

The second-most-frequent mechanism of acquired resistance corresponds to the activation of alternative pathways. In this regard, MET oncogene amplification (a copy number of the MET gene ≥ 5 or a MET/CEP7 ratio of ≥2) is the most-important one and represents about 5–22% of cases, regardless of the EGFR-TKI generation or line of therapy [92,119,120]. MET is a tyrosine kinase receptor that is activated by the hepatocyte growth factor (HGF). This leads to the activation of the downstream AKT pathway, resulting in cell proliferation [121]. MET amplification results in receptor overexpression with a continuous ErbB3-AKT signalling, which is kept despite the blockade of EGFR [122]. 

MET amplification is usually seen concurrently with EGFR exon 19 deletion, and it has been detected with or without the loss of the T790M mutation [92,119]. Moreover, MET amplification was observed in association with the C797S EGFR mutation in 7% of cases in the AURA3 trial, but also with CDK6 or BRAF amplification [96]. In some cases, it was reported concurrently with the EGFR mutation before treatment [123].

Moreover, more than 20 oncogenic mutations of MET have been detected, and most of them are germline. However, MET mutations are rare (P97K/Q, I865F) in NSCLC with the most-common ones that affect the semaphorin domain (avoiding the growth factor binding), the juxtamembrane domain (altering the actin cytoskeleton of the cell), and the TK domain (with the consequent constitutive activation of the receptor, even in the absence of its ligand) [101,103,124,125,126].

The HER2 gene encodes the ErbB2 receptor tyrosine kinase [127]. It is responsible for EGFR-TKI resistance by means of alternative activation via the MAPK or PI3K pathway. Although ErbB2 amplification occurs only in 1% of untreated lung adenocarcinoma, it is responsible for 12% of cases of acquired resistance to first-generation TKIs and 5% to second-line treatment with osimertinib, mutually exclusive with the T790M mutation in both cases [128,129].

Furthermore, 2% of NSCLC patients have ErbB2 mutations at exon 20 encoding for the kinase domain of the receptor [128]. The ErbB2 receptor has a strong kinase activity, although a ligand-binding domain has not been identified; therefore, it is activated following the formation of heterodimers with the other members of EGFR family members [130]. On these bases, the wild-type state of ErbB2 is sensible to EGFR-TKIs in consideration of the EGFR-mediated activation of ErbB2. On the other hand, the mutated state of ErbB2 regarding the kinase domain makes it EGFR-independent and become responsible for resistance to EGFR-TKIs. 

##### Rare Gene Mutations 

Other rare gene mutations (<1%) have been reported. 

KRAS G12S, G12D, G13D, Q61R, and Q61K were described as resistance mutations to second-line osimertinib [23,61,88,96,101]. 

An in vitro study documented NRAS mutations (and the novel E63K) in NSCLC EGFR-mutated cell lines resistant to first-, second-, and third-generation TKIs [131]. Other experimental data reported that the combination of osimertinib with selumintinib prevents EGFR-TKI resistance [132,133,134]. 

BRAF V600E was another resistance mutation to second-line treatment that has been identified in 3% of cases in cfDNA [96], concurrently or not, with the T790M mutation [98,135]. An in vitro study documented BRAF V600E NSCLC cell lines as resistant to osimertinib and sensible to the combination of osimertinib with encorafenib (BRAF inhibitor) [135].

PI3KCA mutations/amplifications and PTEN loss lead to an increased PI3K signaling [110]. They are usually present at baseline concurrently with some other driver mutations in NSCLC, although PI3KCA mutations or amplification were also reported at progression after gefitinib and erlotinib in 3–5% of cases [110]. On the other hand, some PI3KCA mutations such as E454K, E452K, R88Q, N345K, and E418K were identified at progression after second-line osimertinib in 4–11% of patients [23,136]. Moreover, an in vitro study showed the PI3KCA E545K mutation as involved in osimertinib resistance [23]. In the AURA3 trial, the co-existence of PI3KCA amplification and HER2 amplification was reported through NGS analysis of cfDNA [96]. PTEN loss was also identified as an acquired mechanism of resistance [137].

AXL gene upregulation with the consequent protein overexpression was described as another mechanism of acquired resistance to EGFR-TKIs [110,138].

Amplifications of genes regulating the cell cycle such as cyclin D1, cyclin D2, cyclin-dependent kinase N2A, cyclin E1, and CDK4/6 were reported in 12% of cfDNA samples after second-line third-generation EGFR-TKI treatment [96].

##### Gene Fusions 

Gene fusions were reported after first-generation EGFR-TKIs very rarely and in 3–10% of patients after second-line osimertinib [139]. They were seen concurrently with the EGFR C797S mutation, MET amplification, or BRAF mutation [96]. The most-common gene fusions regard RET (46%), in particular RET-ERC1, RET-CCDC6, RET-RUFY2, and RET-NCOA4 [101,140]. Second are ALK fusions (26%) such as ALK-EML4 after second-line osimertinib [141], ALK-SPTBN1, and ALK-PLEKHA7 after first-line osimertinib [116,142]. Third are NTRK1 fusions (16%) including NTRK1-TPM3, which has been described in association with the EGFR Del19 and T790M mutations. Fourth are FGFR3 fusions (11%) such as FGFR3-TACC3, which was reported in combination with the EGFR Del19, C797X, and T790M mutations [96]. 

Other gene fusions have been described regarding ROS1 (ROS1-GOPC), MET (MET-SPECC1L), and BRAF (BRAF-ESYT2, BRAF-AGK, BRAF-BAIAP2L1, BRAF-PCBP2, BRAF-TRIM24, and BRAF-PJA2) [23,139,143,144].

##### Activation of Cell Receptors

The activation of the insulin-like growth factor 1 receptor (IGF-1R) seems to be related to EGFR-TKI resistance as shown in in vitro studies on lung cancer cell lines resistant to gefitinib or erlotinib [145]. A possible mechanism could depend on IGF-1R activation as a consequence of the heterodimerisation with EGFR after erlotinib treatment [145]. This event leads to the transmission of extracellular survival signals to downstream intra-cellular factors such as MAPK and AKT. On this basis, the combination of IGF-1R inhibitors such as AG1024, α-IR3, or R1507 with EGFR-TKIs might be a new strategy to overcome the resistance [146].

The activation of FGFR2 and 3 could also play a pivotal role in the drug resistance of cancer cells [147].

##### Phenotypic Transformation

In rare cases, a histological transformation from NSCLC (adenocarcinoma) to SCLC was observed [92]. In particular, this event was reported for some patients under EGFR-TKI treatment of first-, second-, and third-generations [101,106,148]. Therefore, it is considered a mechanism of acquired resistance.

A possible explanation of this phenomenon could be the existence of one shared multipotent stem cell for both adenocarcinoma and SCLC. On the other hand, other hypotheses suggest the development of SCLC through the expansion of minor pre-existent cells under the selection pressure of EGFR-TKIs or transdifferentiation from the adenocarcinoma cells [149]. 

A genomic study showed that transformed SCLC tumour samples retain the original EGFR-activating mutation detected at the baseline biopsy sample. This suggests that the transformed phenotype was not generated by de novo clones, but rather by cancer cells [92]. 

EMT plays a pivotal role in tumour transformation, invasion, and metastasis [150,151]. It is characterised by an important remodelling of the cell cytoskeleton due to the loss of polarity and cell–cell contacts by the epithelial cell layers. Therefore, these cells acquire a mesenchymal phenotype through the loss of E-cadherin and overexpression of mesenchymal proteins such as fibronectin, vimentin, and N-cadherin [152]. EMT is also characterised by AXL upregulation, which is considered a novel mechanism of acquired resistance to EGFR-TKI in NSCLCs. In this regard, pre-clinical studies documented that, on the one hand, the pharmacological inhibition of AXL reduced tumour cells’ proliferation and invasion and, on the other hand, increased cancers cells’ chemosensitivity [138].

The activation of the Hedgehog (Hh) pathway is implicated in tumourigenesis, metastatisation, and treatment resistance in various types of human tumours. Recent findings about EMT documented that gene amplification of SMO, a Hh receptor, is another mechanism of acquired resistance to first-generation EGFR-TKIs in EGFR-mutant NSCLC cells [153]. In particular, preclinical results showed that Hh-mediated EGFR-TKI resistance matched with the mesenchymal transformation of EGFR-mutated NSCLC cells [154]. In addition, these preclinical data were confirmed by the results of clinical studies on patients with EGFR-mutant NSCLC under treatment with EGFR-TKIs. In detail, the authors noticed the co-amplification of SMO and MET genes in tumour samples taken as the clinical evidence of EGFR-TKI resistance in 2 of the 16 patients. 

Preclinical models of acquired resistance to EGFR-TKIs in EGFR-mutated NSCLC cells showed that the concomitant inhibition of both SMO and MET led to significant antiproliferative and proapoptotic effects, as well as the loss of the mesenchymal phenotype, suggesting new combination strategies [154].

These data confirmed that the upregulation of the Hh pathway results in EGFR-TKI resistance through EMT induction, while the inhibition of this signalling pathway increased EGFR-TKI sensitivity [154].

## 3. Future Perspectives

**Figure 3:**
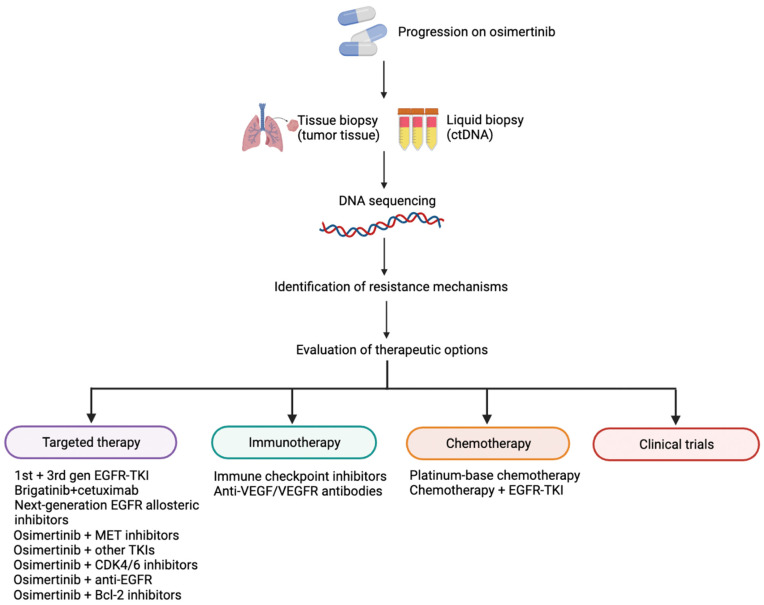
Summary of the procedures after osimertinib resistance. Tissue biopsy and/or liquid biopsy should be performed to identify the mechanisms of resistance to guide treatment. Therapeutic strategies are comprised of targeted therapy, chemotherapy, and immunotherapy or combination regimens. In the case of a lack of valid targets, clinical trials should be considered.

Cancer’s heterogeneity favours the occurrence of molecular resistance mechanisms, hence the difficulty in developing new effective treatment strategies. On the bases of the known resistance mechanisms, platinum-based chemotherapy, targeted therapies, and immunotherapy, or their combinations (Table 4), correspond to the potential therapeutic regimens for patients who progressed on EGFR-TKIs. The reassessment of tumour genetic alterations through tissue biopsy and/or liquid biopsy becomes of supreme importance to define the mechanisms of resistance and to guide the therapeutic strategy. Indeed, a specific treatment will be administered to patients if effective therapies currently exist. Conversely, preclinical studies and clinical trials will be considered to offer new therapeutic strategies. Figure 3 summarises the procedures after the occurrence of resistance.

### 3.1. Other Third-Generation EGFR-TKIs

In addition to osimertinib, other third-generation EGFR-TKIs have been evaluated in NSCLC patients with T790M and EGFR-activating mutations. 

Almonertinib has an optimised structure compared to osimertinib. It has been tested in a phase 2 study on 244 NSCLC patients with EGFR T790M who experienced PD to the previous EGFR-TKI line. The results showed a DCR of 93.4% and an ORR of 68.9%, as well as a PFS of 12.3 months. Moreover, this drug provided an important encephalic response rate for those patients with CNS metastases: the DCR and ORR were 91.3% and 60.9%, respectively, while CNS PFS was 10.8 months [170]. 

Alflutinib inhibits both EGFR-sensitive mutations and acquired EGFR-T790M-positive NSCLC. This drug led to an ORR of 73.6% and mPFS of 7.6 months in a phase 2b in which patients with EGFR-T790M-mutated NSCLC were enrolled [171]. 

Lazertinib is another third-generation EGFR-TKI that showed an important systemic and intracranial activity on EGFR-T790M-positive NSCLC patients in a phase 1/2 study [172]. Currently, a phase 3 clinical trial is ongoing to compare lazertininb and gefitinib as a first-line therapy in NSCLC patients with EGFR-sensitising mutations. 

Abivertinib is an irreversible third-generation EGFR-TKI that selectively targets EGFR-sensitising mutations and the T790M mutation through the formation of a covalent bond with C797 in the ATP-binding pocket. In a phase 2 clinical trial, it led to a DCR of 88.0% and an ORR of 52.2% [173,174]. 

Nazartinib is a covalent and irreversible EGFR-TKI targeting the exon 19 deletion, L858R, and T790M mutations. Preliminary results from a multicentre, open-label, phase 1/2 trial reported an ORR of 44% and a DCR of 91% with a median duration of response of 9.2 months and an excellent safety profile [175]. Ongoing clinical trials have been evaluating nazartinib combined with capmatinib in patients with advanced NSCLC with EGFR L858R, ex19del, or T790M in various lines of therapy including T790M-negative or treatment-naive patients (NCT02335944). 

Olmutinib has potent inhibitory activity against L858R-/T790M-mutant NSCLC cells (44). A single-arm, open-label, phase 1/2 trial analysed olmutinib for EGFR-T790M-positive patients showing an ORR of 55% and a PFS of 6.9 months [176,177]. However, the safety profile of this drug is unfavourable relative to osimertinib, in particular due to skin toxicity including toxic epidermolysis necrosis and Stevens–Johnson syndrome. 

Naquotinib is a small irreversible TKI that binds at C797 and targets the common EGFR mutations including T790M while sparing wild-type EGFR. This drug was tested in a phase 1 trial as a second-line therapy for T790M-positive patients, showing an ORR of 31% and a median PFS of 6.0 months [178]. On the other hand, the first-line naquotinib resulted in an ORR of 52%, DCR of 94%, and PFS of 11.3 months. However, the phase 3 clinical trial of naquotinib was closed due to limited predicted efficacy and toxicity [177]. 

Mavelertinib has a potent cellular inhibitory activity against EGFR L858R, the EGFR del19 mutation, EGFR L858R/T790M, and EGFR del19/T790M, while sparring WT EGFR. A phase 1 study tested this drug with an ORR of 42.3% and a DCR of 65,4% [176,179,180]. Further analyses will evaluate the efficacy and safety of mavelertininb plus palbociclib, according to preclinical data demonstrating synergy (NCT02349633) [179,180].

### 3.2. Therapeutic Options in T790M-cis-C797S Mutations

As mentioned above, if the C797S and T790M mutations are in trans, the cancer will be sensitive to the combination therapy of first- and third-generation EGFR-TKIs, while if they are in cis, EGFR-TKIs alone or in combination will be ineffective [111,181]. For the latter, alternative treatment strategies have been described. A preclinical study and a case report documented an interesting activity of brigatinib, a dual ALK and EGFR inhibitor, when administered in combination with cetuximab (anti-EGFR antibody), for NSCLC patients with T790M/cis-C797S EGFR mutations [182,183]. Moreover, a retrospective study showed that this combination led to an ORR of 60% and a median PFS of 14 months with respect to an ORR of 10% and a median PFS of 3 months for those patients treated with chemotherapy. Other combinations have been described in some case reports for patients with C797S and T790M mutations in cis with promising results [184,185]. The latter suggest that the combination of EGFR-TKIs with anti-VEGFR might be a promising therapy for this subset of patients. Further clinical trials should evaluate an ICI plus platinum-based doublet chemotherapy since it exhibited efficacy against T790M-cis-C797S [186]. The administration of osimertinib plus first-/second-generation EGFR-TKIs could be effective in cases of in trans C797X and T790M mutations, as well as the first-/second-generation EGFR-TKIs in cases with the C997X and without the T790M mutation [111,181]. 

However, currently, no standard therapeutic regimens are recommended for this set of patients. 

### 3.3. Next-Generation EGFR Allosteric Inhibitors

Various therapeutic strategies have been studied to solve the issue of C797S-mediated resistance to osimertinib. In particular, several next-generation EGFR allosteric inhibitors have been developed [91,187,188,189]. They bind to EGFR at a site away from the tyrosine kinase domain, which bypasses the C797S-mediated resistance mechanism [190]. 

EAI045 selectively changes the space configuration of mutated EGFR and hinders its binding to EGFR ligands. In this way, it blocks the phosphorylation and the downstream signalling pathway, including p-STATs, p-AKT, and p-ERK1/2 [187]. In vitro and in vivo data reported a remarkable synergistic effect on Ba/F3 cells with triple mutants (L858R/ C797S/T790M) from EAI045 plus cetuximab [187,190]. 

Another next-generation EGFR allosteric inhibitor is JBJ-04-125-02, which exhibited, in vitro and in vivo, an increased apoptosis of NSCLC cells with EGFR triple mutants when combined with osimertinib than JBJ-04-125-02 or osimertinib as a single agent [188,191]. 

Unlike EAI045, CH7233163 proved to inhibit the growth of NSCLC cells with the Del19/T790M/C797S triple EGFR mutants [192]. 

BLU-701 is another fourth-generation EGFR-TKI against the C797S-resistance mutation that demonstrated antitumour activity in a PC9-cell-line-derived tumour xenograft model [193,194]. In addition, a phase 1/2 clinical study (SYMPHONY trial) is testing BLU-945 against the EGFR-sensitised mutation/T790M/C797S [195,196,197,198]. 

Despite these new drugs having demonstrated a potent activity on osimertinib-resistant NSCLC cells in preclinical studies, most of them have not been tested in clinical trials yet.

### 3.4. Osimertinib Plus MET Inhibitors

As mentioned above, MET amplification corresponds to the most-frequent EGFR-independent mechanism of osimertinib resistance (5–24%) [23,199,200]. Therefore, some MET-TKIs have been tested in MET-amplification NSCLC patients to overcome this type of resistance to osimertinib. 

In a phase 1b trial (NCT02143466), savolitinib demonstrated an ORR of 33% and a median PFS of 5.4 months when administered with osimertinib [91,155]. The SACHI phase 3 trial is ongoing with the aim to compare this combination with pemetrexed plus platinum. Interestingly, the TATTON study was designed to analyse the safety of osimertinib plus selumetinib (MEK1/2 inhibitor), savolitinib (MET inhibitor), or durvalumab (anti-PD-L1 monoclonal antibody) in advanced EGFR-mutant NSCLC patients who progressed on a previous EGFR-TKI line. The interim results showed that osimertinib combined with savolitinib had acceptable toxicity and favourable antitumour activity [133,155]. Capmatinib, another MET inhibitor, showed an ORR of 27% in combination with gefitinib [91,156]. GEOMETRY-E is an ongoing phase 3 clinical trial testing the combination of capmatinib plus osimertinib compared to platinum–pemetrexed doublet chemotherapy as a second-line therapy for advanced NSCLC [201]. In the INSIGHT trial, tepotinib, a MET inhibitor, in association with gefitinib, documented an mPFS of 4.9 months versus the 4.4 months of the chemotherapy group (HR 0.67, 90% CI 0.35–1.28); mOS was 17.3 months in the combination group versus 18.7 months in the chemotherapy group (15.9–20.7; HR 0.69, 0.34–1.41) [91,157]. 

Amivantanab is a bispecific EGFR and MET antibody that is under evaluation in combination with lazertinib, another third-generation EGFR-TKI, in a phase 3 clinical study [158]. Some case reports and a retrospective analysis revealed important clinical and radiographic responses in EGFR-mutation-positive NSCLC patients treated with crizotinib plus osimertinib [202,203,204].

### 3.5. Osimertinib Plus CDK4/6 Inhibitors

Cyclin-dependent kinase (CDK) inhibitors, such as palbociclib and abemaciclib, act through the reduction of CDK-induced phosphorylation of downstream Rb [205,206,207]. Thus, this class of drug can inhibit the switch from the G1 to S phase of the cell cycle [205]. Experimental data reported as the combination of CDK4/6 inhibitors plus osimertinib led to an increased proportion of cells in the G1 phase and the block of the proliferation of osimertinib-resistant cells [205,206]. In addition, other studies revealed that CDK7 inhibitors might be effective to counter EMT-mediated resistance to osimertinib [208]. Two clinical trials are investigating the clinical efficacy of CDK4/6 inhibitors (abemaciclib or lerociclib) plus osimertinib in EGFR-mutation-positive metastatic NSCLC patients who progressed on osimertinib therapy in NCT04545710 and NCT03455829, respectively. 

### 3.6. EGFR-TKIs Plus Anti-EGFR Antibodies

Preclinical data reported that the combination of the dual inhibition with an EGFR-TKI and anti-EGFR antibody was characterised by an elevated activity on EGFR L858R/T790M erlotinib-resistant tumours [209]. Phase 1b/2 clinical trials evaluated afatinib plus cetuximab, afatinib plus nimotuzumab, or osimertinib plus necitumumab for patients affected by EGFR-mutant NSCLC with acquired resistance to gefitinib or erlotinib, regardless of the EGFR T790M mutation [159,160,210,211]. These studies showed an important clinical activity and a safe profile of toxicity. Furthermore, another study investigated necitumumab, an anti-EGFR monoclonal antibody, in combination with chemotherapy versus chemotherapy alone as the first-line therapy. The results reported a longer OS (11.5 months vs. 9.9 months) for advanced squamous NSCLC patients in the experimental group [161]. Finally, several clinical trials (NCT03944772, NCT04285671, and NCT02496663) are testing the efficacy of necitumumab plus osimertinib in NSCLC with progression on EGFR-TKIs, including osimertinib.

### 3.7. Third-Generation EGFR-TKI Plus Bcl-2 Inhibitor

A phase 1b trial is ongoing with the aim to test osimertinib in combination with navitoclax in 27 patients with disease progression under an anti-EGFR-TKI [162]. Navitoclax is a dual-inhibitor of Bcl-2 and BCL-xL, which induces BIM (a proapoptotic protein from Bcl-2 family components) by decreasing the capacity of BCL-xL to neutralise BIM and facilitates Bax and Bak to initiate caspases, leading to cell death. In the expansion cohort, the ORR was 100% and the PFS was 16.8 months.

### 3.8. Chemotherapy

Despite several resistance mechanisms to osimertinib having been defined, 30–50% of them are still unknown. For the latter, no targeted therapy options exist; therefore, platinum-based chemotherapy is the best choice of treatment, especially in the case of SCLC transformation. In particular, a retrospective analysis reported an important activity of platinum–etoposide for SCLC-transformed patients (ORR of 54%, mPFS of 3.4 months, OS of 10.9 months). No efficacy was observed for those patients that underwent ICIs [212]. Another retrospective study showed that chemotherapy provides a better survival (25.0 versus 11.8 months) than a non-chemotherapy strategy in advanced NSCLC patients who progressed under first-line therapy with Osimertinib and without a molecular target [213]. 

However, it is controversial if the continuing administration of EGFR-TKIs during chemotherapy could provide a benefit. To this end, the IMPRESS phase 3 trial evaluated the efficacy and safety for patients who continued gefitinib in addition to cisplatin and pemetrexed after progression on first-line gefitinib treatment with respect to chemotherapy alone [163,214]. The experimental group experienced a similar PFS (5.4 vs. 5.4 months), but worse OS (13.4 vs. 19.5 months). On the other hand, the NEJ009 phase 3 trial showed that the combination of gefitinib plus carboplatin and pemetrexed led to a longer PFS (20.9 versus 11.9 months) and OS (49.0 vs. 38.5 months) than gefitinib as a single agent [164,215]. Therefore, the association therapy with gefitinib and chemotherapy provides opposite results in the different treatment settings. This is probably due to gene alterations in NSCLC patients after therapy with EGFR-TKIs. However, limited clinical data have been published about the efficacy of continuing osimertinib in addition to chemotherapy for those patients who previously received osimertinib as a first-line therapy. A randomised phase 2 clinical trial evaluated osimertinib plus carboplatin–pemetrexed compared to osimertinib alone for EGFR-mutated NSCLC patients with T790M in the second-line setting. The results reported a median PFS of 15.8 months versus 14.6 months and an ORR of 71.4% versus 53.6% in the control group and experimental group, respectively [165]. On the other hand, the combination of osimertinib with chemotherapy did not provide a survival benefit in the second-line setting, similar to gefitinib. Currently, some clinical studies are ongoing to further confirm these results. To be specific, the FLAME and COMPEL trials are enrolling EGFR-mutant NSCLC patient to compare osimertinib plus chemotherapy with osimertinib alone. Furthermore, the FLAURA2 phase 3 trial is evaluating first-line osimertinib plus platinum-based chemotherapy compared to osimertinib as a single agent [56].

### 3.9. Immunotherapy

Several phase 3 clinical trials reported very favourable results derived from the administration of ICIs for NSCLC patients without targetable mutations and many other cancers [216]. On the other hand, immunotherapy led to a limited benefit in NSCLC patients harbouring EGFR alterations [217,218]. In this regard, the IMMUNOTARGET registry revealed that immune monotherapy determined a low ORR and PFS among EGFR-mutated NSCLC patients, 12% and 2.1 months, respectively [219]. In addition, the single agent pembrolizumab provided limited efficacy in the subset of EGFR-mutated NSCLC patients who did not previously receive EGFR-TKI therapy, even in the case of PD-L1 expression of more than 50% [220]. A higher incidence of hyperprogressive disease (HPD) was observed with the administration of ICI monotherapy in NSCLC patients harbouring EGFR alterations [221,222]. On these bases, ICI monotherapy is not considered an appropriate therapeutic option for this subset of patients. The explanation for this lower sensitivity to anti-PD-1/-PD-L1 treatment has not been well defined yet, but it might be correlated with the low tumour mutational burden (TMB) of NSCLC with targetable mutations, with consequent weak immunogenicity [223]. 

In consideration of the limited benefit of ICI monotherapy in EGFR-mutated NSCLC patients, combination strategies have been evaluated. Recently, experimental data showed that PD-L1 expression and increased tumour-infiltrating lymphocyte density in the tumour microenvironment are correlated with the EGFR T790M mutation and response to the anti-PD1 antibody [224,225]. These results led to the evaluation of PD-1 blockade plus EGFR-TKI treatment for EGFR-driven NSCLC. In detail, the combinations of nivolumab and erlotinib and of durvalumab and osimertinib were associated with an important efficacy, but, at the same time, severe toxicity including interstitial pneumonitis, interstitial lung disease, and liver enzyme elevation [166,167,226,227,228,229,230,231,232]. Therefore, this type of combination did not demonstrate promising results due to the high incidence of Grade 3/4 adverse events (AEs). Interestingly, Schoenfeld et al. revealed that sequential ICI followed by osimertinib was associated with severe immune-associated AEs, unlike patients who received osimertinib followed by ICI [229]. Therefore, caution should be taken when osimertinib is administered to patients who recently received ICIs. 

Recent studies are evaluating the combination of immunotherapy plus chemotherapy and antiangiogenetic agents for EGFR-mutant NSCLC patients intending to increase sensitivity to immunotherapy by modulating the tumour microenvironment [91]. In this regard, the IMpower150 phase 3 trial tested the combination of atezolizumab, carboplatin, paclitaxel, and bevacizumab with respect to the same chemotherapy plus bevacizumab, in non-squamous NSCLC patients, including those with *EGFR* mutations who could have received prior EGFR-TKIs. This study showed a significant improvement in OS and the PFS for the experimental group [168]. Similarly, promising results derived from another clinical trial in which the combination of atezolizumab, bevacizumab, carboplatin, and pemetrexed was evaluated for advanced EGFR-mutated NSCLC patients who progressed on an EGFR-TKI [233]. The ORIENT-31 is a phase 3 clinical trial that is evaluating sintilimab plus IBI305 (bevacizumab biosimilar), cisplatin, and pemetrexed compared to chemotherapy alone in EGFR-mutated NSCLC patients after EGFR-TKI failure. Interim results revealed a significantly longer PFS (6.9 months vs. 4.3 months) for the experimental group [169]. 

Other combination treatments are under investigation.

## 4. Conclusions

EGFR-TKIs are considered the gold standard treatment for patients affected by EGFR-mutated advanced NSCLC since these medicines have provided a remarkable improvement in clinical outcomes. Although osimertinib is the first-line therapy for these patients thanks to its potent activity and favourable safety profile, as well as early-generation EGFR-TKIs, resistance inevitably occurs due to various mechanisms. The reassessment of molecular alterations thorough tissue biopsy and/or liquid biopsy become of supreme importance to define the mechanisms of resistance and to guide therapeutic strategy. Indeed, targeted therapies, immunotherapy, and chemotherapy are providing interesting results for NSCLC patients harbouring EGFR mutations and with osimertinib resistance. Moreover, an increasing number of clinical trials is evaluating the efficacy and safety of several combination regimens. 

Resistance to EGFR-TKIs hampers the correct management of EGFR-mutated NSCLC patients; therefore, the development of novel drugs to overcome this issue is fundamental. In addition, further studies are necessary to deepen knowledge about resistance mechanisms to osimertinib since a considerable proportion of them are still unknow. Indeed, in-depth knowledge of the resistance mechanisms would make it possible to individualise the treatment strategies, providing an improvement in the quality of life and a survival benefit of NSCLC patients.

## Figures and Tables

**Table 1:** EGFR mutations: common, uncommon, and other alterations.

EGFR Mutations	Specific Alteration		Frequency	Clinical Characteristics	Response to EGFR-TKIs		
					Gefitinib/Erlotinib	Afatinib	Osimertininb
Common	Exon 19 deletion		45–50%	Adenocarcinoma Female gender Never smoker status Asian ethnicity	Sensitive	Sensitive	Sensitive
	Exon 21 L858R point mutation		37–40%		Sensitive	Sensitive	Sensitive
Uncommon or rare	Exon 18	G719X	3%	Adenocarcinoma Male gender Smoker history Asian ethnicity	Intermediate	Sensitive	Inter./sensitive
		E709X	0.3%		E709K Intermediate	Sensitive	Sensitive
		Del18	0.3%		Intermediate	Sensitive	Sensitive
	Exon 19	Ins19	<0.6%		Intermediate	Inter./Sens.	Inter./Sens.
	Exon 20	Ins20	>5.8%		Resistant	Resistant/Sens.	Inter./Sens.
		S768I	<1.5%		Intermediate	Inter./Sens.	Sensitive
	Exon 21	L861Q	0.9–3%		Intermediate	Inter./Sens.	Inter./Sens.
Other EGFR alterations	Complex mutations are a combination between: - common and rare mutations - rare and rare mutations - common and common mutations		5–15%	Depend on the specific combination of mutations	Depend on the specific combination of mutations		
	Co-mutations are combinations of EGFR mutation(s) with one or more mutations of another gene (tumour suppressor gene or oncogene)		3–6%				
	Subclonal mutations: the presence of the specific mutation only in a small percentage of tumour cells		Variable				

**Table 2:** Clinical trials evaluating all generations of EGFR-TKIs.

Generation EGFR-TKI	Clinical Trail	EGFR Status	Comparison	Results
First-generation	NEJ002 [28,29]	Common mutations	Gefitinib vs. carboplatin plus paclitaxel	* PFS: 10.8 vs. 5.4 mo. ** OS: 27.7 vs. 26.6 mo.
	IPASS [30,31]	Common mutations	Gefitinib vs. carboplatin plus paclitaxel	* PFS: 9.5 vs. 6.3 mo. ** OS: 18.8 vs. 17.4 mo.
	WJTOG3405 [32,33]	Common mutations	Gefitinib vs. cisplatin plus docetaxel	* PFS: 9.2 vs. 6.3 mo. ** OS: 34.9 vs. 37.3 mo.
	OPTIMAL [34,35]	Common mutations	Erlotinib vs. gemcitabine plus carboplatin	* PFS: 13.1 vs. 4.6 mo. ** OS: 22.8 vs. 27.2 mo.
	ENSURE [36]	Common mutations	Erlotinib vs. gemcitabine plus cisplatin	* PFS: 11 vs. 5.5 mo. ** OS: 26.3 vs. 25.5 mo.
	EUTARC [37]	Common mutations	Erlotinib vs. cisplatin plus docetaxel or gemcitabine	* PFS: 9.7 vs. 5.2 mo. ** OS: 22.8 vs. 27.2 mo.
	CONVINCE [38]	Common mutations	Icotinib vs. cisplatin plus pemetrexed	* PFS: 11.2 vs. 7.9 mo. ** OS: 30.5 vs. 32.1 mo.
Second-generation	LUX-Lung 3 [39]	Common mutations	Afatinib vs. cisplatin plus gemcitabine or pemetrexed	* PFS: 13.6 vs. 6.9 mo. ** OS: 28.2 vs. 28.2 mo.
	LUX-Lung 6 [40]	Common mutations	Afatinib vs. cisplatin plus gemcitabine or pemetrexed	* PFS: 11 vs. 5.6 mo. ** OS: 23.1 vs. 23.5 mo.
	ARCHER 1050 [40,41]	Common mutations +/− T790M	Dacotinib vs. gefitinib	* PFS: 14.7 vs. 9.2 mo. ** OS: 34.1 vs. 27 mo.
Third-generation	AURA3 [42]	T790M mutation	Osimertinib vs. cis/carboplatin plus pemetrexed after first-line EGFR-TKI therapy	* PFS: 10.1 vs. 4.4 mo. ** OS: 26.8 vs. 22.5 mo.
	FLAURA [26]	Common mutations	Osimertinib vs. gefitinib or erlotinib	* PFS: 18.9 vs. 10.2 mo. ** OS: 38.6 vs. 31.8 mo.
	ADAURA [43]	Common mutations	Osimertinib for 3 years as adjuvant therapy	* 4ys-DFS: 73% vs. 38%
EGFR-TKIs specific for Ins20	ZENITH20-2 [44]	Exon 20 insertions	Poziotininb	ORR: 27.8% DCR: 70% PFS: 5.5 mo.
	EXCLAIM [45]	Exon 20 insertions	Mobocertinib	ORR: 43% PFS: 7.3 mo.

*: statistically significant difference; **: no statistically significant difference.

**Table 3:** Clinical trials evaluating EGFR-TKI treatment combinations.

Clinical Trail	EGFR Status	Comparison	Results
IFCT-1503 ACE-Lung [47]	Exon 19 deletions, L858R, G719X, L861Q, and S768I mutations or exon 19 insertions	Afatinib plus cetuximab vs. afatinib	Ended for futility
BELIEF [52]	Common mutations +/− T790M	Erlotinib plus bevacizumab	PFS in ITT: 13.2 mo. PFS in T790M-positive group: 16 mo. PFS in the T790M-negative group: 10.5 mo.
JO25567 [53]	Common mutations	Erlotinib single agent or with bevacizumab	* PFS: 16 vs. 9.7 ** OS: 47.0 vs. 47.4 mo.
RELAY [54]	Common mutations	Erlotinib plus ramucirumab vs. erlotinib	* PFS: 19.4 vs. 12.4 mo. OS: immature data
FLAURA2 [56]	Common mutations +/− T790M	Chemotherapy plus osimertinib	Ongoing

*: statistically significant difference; **: no statistically significant difference.

**Table 4:** Early-phase combinations for EGFR-mutated NSCLC patients with osimertinib resistance.

Clinical Trail	Phase	Arm(s)	Endpoint
NCT02143466 [155]	1b	Osimertinib + savolitinib	ORR: 33% PFS: 5.4 mo.
SAVANNAH	2	Osimertinib + savolitinib	ORR (ongoing)
SACHI	3	Osimertinib + savolitinib vs. pemetrexed + platinum	PFS (ongoing)
TATTON [133]	1b	Osimertinib plus: selumetinib (MEK1/2 inhibitor), savolitinib (MET-TKI), or durvalumab (anti-PD-L1).	Safety and tollerability
INC280 [156]	1b/2	Capmatinib plus gefitinib	ORR: 27%
GEOMETRY-E	3	Capmatinib + osimertinib vs. pemetrexed + platinum	DLT, PFS (ongoing)
INSIGHT [157]	1b/2	Tepotinib plus gefitinib vs. standard platinum chemotherapy	PFS: 4.9 vs. 4.4 mo. OS: 17.3 vs. 18.7 mo.
MARIPOSA [158]	3	Amivantanab + lazertinib vs. osimertinib	PFS (ongoing)
NCT04545710	2	Osimertinib + abemaciclib	PFS
NCT03455829	1/2	Osimertinib + lerociclib	DLT, RP2D, AEs, PFS
NCT01090011 [159]	1b	Afatinib + cetuximab	ORR: 30% PFR: 5 mo.
NCT01861223 [160]	1b/2	Afatinib + nimotuzumab	ORR: 23% PFS: 4.3 mo. OS: 11.7 mo.
SQUIRE [161]	3	Necitumumab + gemcitabine + cisplatin vs. gemcitabine + cisplatin	OS: 11.5 vs. 9.9 mo.
NCT02520778 [162]	1b	Osimertinib plus navitoclax	ORR: 100% PFS: 16.8 mo.
IMPRESS [163]	3	Gefitinib + cisplatin + pemetrexed vs. cisplatin + pemetrexed	OS: 13.4 vs. 19.5 mo. PFS: 5.4 vs. 5.4 mo.
NEJ009 [164]	3	Gefitinib vs. gefitinib + carboplatin+ pemetrexed	OS: 49 vs. 38.5 mo. PFS: 20.9 vs. 11.9 mo.
jRCTs071180062 [165]	2	Osimertinib + carboplatin + pemetrexed vs. osimertinib	PFS: 15.8 vs. 14.6 mo. ORR: 71.4% vs. 53.6%
FLAME	2	Osimertinib plus chemotherapy vs. osimertinib	PFS (ongoing)
COMPEL	2	Osimertinib plus chemotherapy vs. osimertinib	PFS (ongoing)
FLAURA 2 [56]	3	Osimertinib + platinum–pemetrexed vs. platinum–pemetrexed	PFS (ongoing) OS (ongoing)
CheckMate 012 [166]	1	Nivolumab + erlotinib	ORR:15%
CAURAL [167]	3	Osimertinib + durvalumab vs. osimertinib	AEs
IMpower150 [168]	3	Atezolizumab plus bevacizumab plus carboplatin plus paclitaxel vs. bevacizumab plus carboplatin plus paclitaxel	OS: 19.5 vs. 14.7 mo.
ORIENT-31 [169]	3	Sintilimab + bevacizumab biosimilar IBI305 + chemotherapy vs. chemotherapy alone	PFS: 6.9 vs. 4.3 mo.

## Data Availability

Not applicable.
